# Pain Modulates Responses to Emotional Stimuli

**DOI:** 10.3389/fpsyg.2020.595987

**Published:** 2020-11-09

**Authors:** Wanchen Li, Peiyi Liu, Yuanyan Hu, Jing Meng

**Affiliations:** ^1^Key Laboratory of Applied Psychology, Chongqing Normal University, Chongqing, China; ^2^School of Education, Chongqing Normal University, Chongqing, China; ^3^Key Laboratory of Emotion and Mental Health, Chongqing University of Arts and Sciences, Chongqing, China

**Keywords:** pain, emotional stimuli, sad scenes, negative emotional stimuli processing, attentional processing

## Abstract

Pain and emotion are common subjective experiences that play vital roles in daily life. Pain has been clinically confirmed to increase depressive mood. However, little is known about how pain modulates cognitive emotional judgment processing. A better understanding of this may help explain the effect of pain on the development of depressive moods. We recruited 30 adult participants to test their responses to pictures of scenes (Experiment 1) and faces (Experiment 2) that represented happy, neutral, and sad emotions, while experiencing painful (induced via topical capsaicin cream) and control (hand cream) treatments. Results showed that participants in the painful condition showed lower accuracy to emotional scene stimuli and longer reaction times to both emotional scene and face stimuli, relative to the control condition. In addition, the difference values of the reaction times between the painful and control conditions were larger for sad scenes than for happy or neutral scenes. These results suggest that pain alters attentional processing of emotional stimuli, especially with regards to sad scene stimuli, which may explain how painful stimuli affect the development of depressive moods.

## Introduction

Pain has evolutionary significance to humans, whereby the behaviors evoked by pain are critical for human survival (Wang et al., 2019). Recently, the International Association for the Study of Pain (IASP) revised pain as “an unpleasant sensory and emotional experience associated with, or resembling that associated with, actual or potential tissue damage” (Raja et al., 2020).

Pain and emotion are closely related. From a theoretical perspective, pain can be defined as a type of unpleasant emotional experience and includes the feelings of depression and sadness (Mokhtari et al., 2019). Particularly, the motivational-affective dimension of pain is closely linked with emotion (Melzack and Casey, 1968). From a neuropsychological perspective, similar brain regions represent both pain and emotion. For example, the medial frontal cortex (including the anterior midcingulate cortex; Kragel et al., 2018), the midbrain periaqueductal gray (Buhle et al., 2013), and the hippocampus (Mokhtari et al., 2019) are involved in both pain and negative emotions, suggesting that the experience of pain influences the processing of negative emotions.

There are bidirectional influences of pain and emotion processing (Reicherts et al., 2013). There has been substantial behavioral and neurobiological research on the effects of emotional stimuli on pain processing. For instance, Lu et al. (2019) identified that positive emotional stimuli from music could reduce the unpleasantness of pain. Further, Willoughby et al. (2002) used a laboratory-induced depressive mood to examine its effects on responses to pain, which revealed that a depressive mood lowered pain tolerance and increased pain catastrophizing. In contrast, the way that pain might modulate emotion processing has been rarely investigated. Clinical evidence has indicated that patients with pain often experience emotional disturbances, especially depressive moods (Hawker et al., 2011; Craig et al., 2013), and experience dysfunctional processing of emotional stimuli (Bartley et al., 2008; Rosselló et al., 2015; Giel et al., 2018), which hints at a potential effect of pain on emotion processing. One study that required participants to assess emotional scenes under painful or innocuous electrical shocks showed that painful stimuli significantly reduced the emotional ratings of pleasant pictures and decreased visually evoked brain responses to pleasant emotional stimuli (Godinho et al., 2008). Similarly, in a study by Gerdes et al. (2012), where pressure pain was experimentally induced during the viewing of emotional faces, researchers observed that painful stimuli slowed individuals’ facial muscle responses to happy emotional faces, while emotional ratings of the pictures remained the same. However, Wieser et al. (2012) reported that emotional ratings and early emotion discrimination in response to happy emotional faces did not change with tonic pressure pain. With regard to the processing of negative emotional stimuli, the aforementioned studies reported no significant influences of pain on the processing of negative emotional stimuli, regardless of the type of pain stimuli used (Godinho et al., 2008; Gerdes et al., 2012; Wieser et al., 2012). However, another study found that thermal pain enhanced the processing of negative emotional faces (Reicherts et al., 2013). This inconsistency may be the result of differences in the modalities used to induce pain or the duration of pain. For example, Godinho et al. (2008) used a brief electrical shock, which only appeared at the beginning and end of each block. Similarly, the pressure stimuli used in the studies by Gerdes et al. (2012) and Wieser et al. (2012) also only lasted for several seconds. In contrast, the thermal pain stimuli used in the study by Reicherts et al. (2013) had a considerably longer duration that lasted throughout the experiment.

Laboratory-induced pain allows experimental control and enables causal inferences to be drawn (Bresin et al., 2017). Cold pressor pain (e.g., Hollin and Derbyshire, 2009), pressure pain (e.g., Wieser et al., 2012), electrical shock pain (e.g., Godinho et al., 2008), and thermal pain (e.g., Reicherts et al., 2013) are commonly used approaches to experimentally induce pain. However, the limitation of these modalities is that they are often short in duration. Recently, according to the heat/capsaicin sensitization model (Modir and Wallace, 2010), capsaicin has been used to induce a moderate level of sustained painful sensations (Wang et al., 2018, 2019). Furthermore, capsaicin can reproduce the common symptoms of neuropathic pain (Shenoy et al., 2011). In healthy participants, pain induced by capsaicin is reproducible in repeated experiments (Harding et al., 2001). Therefore, we selected capsaicin to induce pain in the current study.

Top-down attention could affect the processing of negative emotional stimuli (Meng et al., 2019), and the attentional effect of pain has now been well documented. According to the cognitive-affective model of pain (Eccleston and Crombez, 1999), intense pain has an interruptive function that draws the attention of the person experiencing the pain. Several findings have supported this notion (Ochsner and Gross, 2005; Wieser et al., 2012; Reicherts et al., 2013). Research has demonstrated that this attentional effect of pain can weaken the processing of emotional stimuli (Wieser et al., 2012). In particular, altered attention toward negative emotional stimuli has been observed in patients experiencing pain frequently. In the study of Duschek et al. (2014), patients experiencing pain had longer reaction times in response to negative emotional words in the Stroop task, which suggests an attention bias for negative emotional stimuli. In a study that used eye tracking technology to assess attentional processing of emotional stimuli in patients with back pain, they showed an attentional bias for negative stimuli, which was expressed as more fixation, larger pupil diameter, longer average fixation duration, and faster first fixation to negative stimuli (Franklin et al., 2018). Fashler and Katz (2016) found that attentional biases toward negative stimuli in individuals experiencing pain appeared primarily in the late phase of attention. Based on these findings, it appears that pain can induce dysfunctional processing of emotional stimuli. The current study aims to examine whether this effect on emotional stimuli processing also occurs in healthy people who do not experience pain frequently.

Images of faces include more emotional information, while pictures of scenes include more perceptual information (Li et al., 2019). Although emotional scene and face stimuli are commonly utilized to experimentally induce emotional states (Godinho et al., 2008; Gerdes et al., 2012; Wieser et al., 2012; Reicherts et al., 2013), there are strikingly distinct patterns of physiological and neurobiological responses between the two types of stimuli. Startle amplitudes and orbicularis oculi responses to positive scene stimuli have shown to be larger than those of positive face stimuli, while heart rate deceleration and skin conductance responses to negative emotional scenes have shown to be greater than those of negative emotional faces (Alpers et al., 2011). Larger right amygdala responses have been observed for emotional faces, while larger left amygdala responses were seen for emotional scenes (Hariri et al., 2002). The processing of emotional faces has also been associated with activations in the anterior fusiform gyrus and middle temporal gyrus, while emotional scenes have shown to activate the lateral occipital cortex, pulvinar, medial dorsal nucleus of the thalamus, extrastriate cortex, and inferior frontal gyrus (Keightley et al., 2010; Sabatinelli et al., 2011). Additionally, both the valence and arousal of emotional scenes and faces can differ (Alpers et al., 2011). Individuals also show different behavioral and neural responses to faces and scenes (Li et al., 2019). In view of these differences, the different modulatory effects of pain on emotional scene stimuli and emotional face stimuli need to be examined in one study. To explore the effects of pain on different emotional stimuli, we decided to use emotional scenes as experimental stimuli in Experiment 1 and emotional faces in Experiment 2.

In an effort to gain a better understanding of the modulatory effect of pain on emotion, we conducted two experiments using emotional scenes (Experiment 1) and faces (Experiment 2) to examine the changes in response to emotional stimuli following laboratory-induced pain. The motivational priming hypothesis (Lang, 1995) assumes that pain may augment the processing of unpleasant stimuli and lessen the processing of pleasant stimuli. Based on this supposition, we hypothesize that (1) pain will prolong attention toward negative (sad) emotional stimuli, and (2) pain will dampen responses to positive (happy) emotional stimuli.

## Materials and Methods

### Participants

Thirty adults (15 females) participated in this study as paid volunteers. All participants were right-handed, aged 21–27 years [mean (M) = 23.47, standard deviation (SD) = 1.74], had normal or corrected-to-normal vision, and had no neurological or psychiatric conditions or chronic pain. Written informed consent was obtained prior to participation. The current study conforms to all provisions of the Declaration of Helsinki and was approved by the local research Ethics Committee of Chongqing Normal University. All procedures were performed in accordance with ethical guidelines and regulations.

### Pain Induction and Assessment

In accordance with previous studies (Wang et al., 2018, 2019), two treatments were applied to the participants. In the painful treatment, 0.1 mL of Capzasin-HP cream (capsaicin 0.1%; CHATTEM, United States) was applied to a 2 cm× 2 cm area on the inside of the left forearm. Then, this area was covered with plastic film to ensure skin contact and heat generation that resulted in a steady and persistent pain sensation. Participants had no prior experience of capsaicin. After the experiment, Capzasin-HP cream was wiped away with tissue paper and soapy water. In the control treatment, an equivalent amount of hand cream was applied to the same area. Pain intensity was assessed using a subjective numerical pain visual analog scale (VAS, 0 = no sensation, 10 = utmost pain imaginable; Carlsson, 1983; Bijur et al., 2001) before treatment (baseline), at 15 min after treatment (pretest), and after the whole study (posttest) for both the painful and control treatments.

**FIGURE 1:**
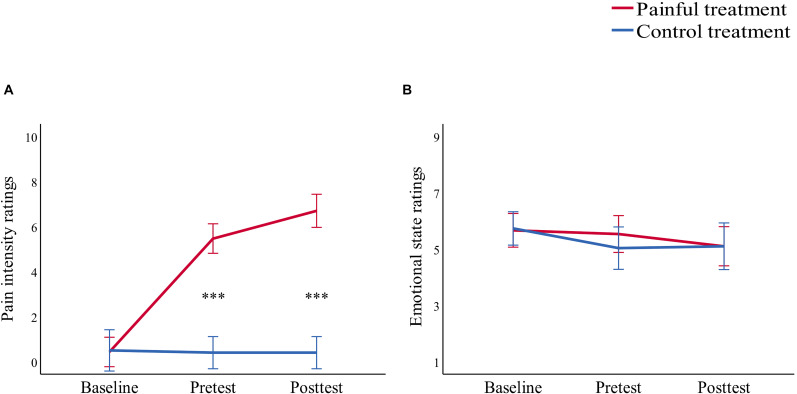
Line charts describing **(A)** pain intensity ratings and **(B)** emotional state ratings before treatment (baseline), 15 min after treatment (pretest), and after the study (posttest) in the painful (red line) and control (blue line) treatments. Data are expressed as mean ± standard error of the mean, ***p < 0.001.

Pain intensity ratings for this study were assessed with two-way repeated-measure analysis of variance (ANOVA) of “treatment” (painful, control) and “time” (baseline, pretest, posttest) (see Table 1 and Figure 1). The interaction of “treatment” × “time” [*F*(1,29) = 255.64, *p* < 0.001, η_p_^2^ = 0.90] indicated that throughout this study, pain intensity ratings of the baseline did not differ significantly in the painful and control treatments (*p* = 0.763). Pain intensity ratings of the pretest and the posttest were both significantly higher in the painful treatment than in the control treatment (both *p* < 0.001). In addition, pain intensity ratings of the posttest (6.73 ± 0.24) were significantly higher than that of the pretest (5.47 ± 0.22, *p* < 0.001) in the painful treatment, demonstrating successful sustained, moderate pain induction from Capzasin-HP cream application.

**TABLE 1:** Summary of the statistical analysis of pain intensity ratings of the two treatments.

	*F*	*p*	η^2^
Treatment	299.86	<0.001	0.91
Time	213.35	<0.001	0.88
Treatment × Time	255.64	<0.001	0.90

*In this study, statistics were obtained using two-way repeated-measure ANOVA with treatment and time. df: (1,29).*

### Stimuli

#### Experiment 1

**FIGURE 2:**
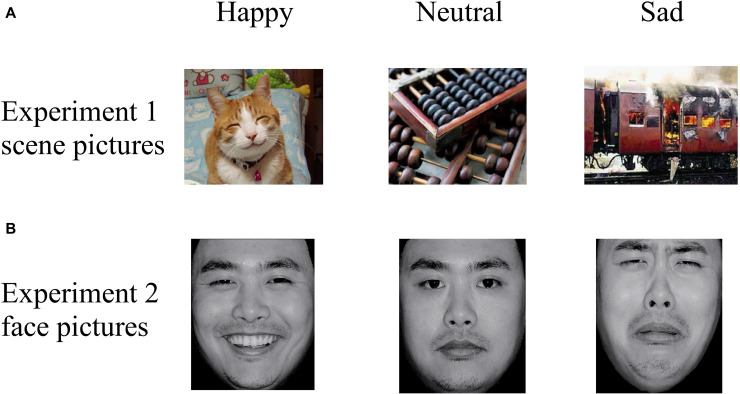
Examples of stimuli in **(A)** Experiment 1 and **(B)** Experiment 2, with examples of happy (left column), neutral (middle column), and sad (right column) pictures. Pictures were selected from the CAPS and the CFAPS that have been previously validated and used in published studies (Bai et al., 2005; Gong et al., 2011; Ma and Zhu, 2014; Wang et al., 2017).

Thirty pictures representing various emotional scenes (10 happy, 10 neutral, and 10 sad) were selected from the Chinese Affective Picture System (CAPS; Bai et al., 2005) that have been previously validated and used in published studies (Zhao et al., 2016; Wang et al., 2017). All scene pictures were in color, as illustrated in the top panel of Figure 2. We recruited 30 undergraduate students (who did not participate in the actual experiment) to assess the valence (1 = very sad, 5 = neutral, 9 = very happy) and arousal (1 = very calm, 5 = neutral, 9 = very exciting) values of the scenes. A one-way repeated ANOVA reported a significant difference among the three categories in “valence” [*F*(2,27) = 61.33, *p* < 0.001, η_p_^2^ = 0.87] and “arousal” [*F*(2,27) = 23.27, *p* < 0.001, η_p_^2^ = 0.72] values (Table 2). The *post hoc* test on the valence showed that it was higher for happy (6.76 ± 0.73) than for neutral (5.11 ± 0.59, *p* = 0.001) and sad (2.71 ± 0.99, *p* < 0.001) scenes, and higher for neutral than for sad scenes (*p* < 0.001). A *post hoc* test showed that the arousal values of the pictures were lower for neutral (3.60 ± 0.41) than for happy (5.26 ± 0.65, *p* < 0.001) and sad (5.85 ± 1.25, *p* < 0.001) scenes, while the latter two categories did not differ significantly (*p* = 0.189).

**TABLE 2:** Summary of one-way repeated ANOVA for valence and arousal values of the emotional stimuli.

		Happy	Neutral	Sad	*F*	*p*	η^2^
Face pictures	Valence	6.76 ± 0.73	5.11 ± 0.59	2.71 ± 0.99	61.33	<0.001	0.87
	Arousal	5.26 ± 0.65	3.60 ± 0.41	5.85 ± 1.25	23.27	<0.001	0.72
Scene pictures	Valence	6.39 ± 0.40	4.82 ± 0.24	3.14 ± 0.43	347.56	<0.001	0.98
	Arousal	5.42 ± 0.34	3.46 ± 0.26	5.18 ± 0.38	118.20	<0.001	0.93

#### Experiment 2

Thirty emotional face pictures (10 happy, 10 neutral, and 10 sad) were chosen from the Chinese Facial Affective Picture System (CFAPS; Gong et al., 2011) that have been previously validated and used in published studies (Ma and Zhu, 2014; Tan et al., 2018). Half of the pictures were of male faces and half were of female faces. Luminance, contrast, and color were matched among the happy, neutral, and sad pictures, and all face pictures were in gray scale, as illustrated in the bottom panel of Figure 2. As with Experiment 1, we recruited 30 undergraduate students (who did not participate in the actual experiment) to assess the valence (1 = very sad, 5 = neutral, 9 = very happy) and arousal (1 = very calm, 5 = neutral, 9 = very exciting) values of the faces. A one-way repeated ANOVA reported a significant difference among the three categories in “valence” [*F*(2,27) = 347.56, *p* < 0.001, η_p_^2^ = 0.98] and “arousal” [*F*(2,27) = 118.20, *p* < 0.001, η_p_^2^ = 0.93] values (Table 2). The *post hoc* test on the valence showed that it was higher for happy (6.39 ± 0.40) than for neutral (4.82 ± 0.24, *p* < 0.001) and sad (3.14 ± 0.43, *p* < 0.001) faces, and higher for neutral than for sad faces (*p* < 0.001). A *post hoc* test showed that the arousal response of the faces was lower for neutral (3.46 ± 0.26) than for happy (5.42 ± 0.34, *p* < 0.001) and sad (5.18 ± 0.38, *p* < 0.001) faces, while the latter two categories did not differ significantly (*p* = 0.158).

### Procedure

**FIGURE 3:**
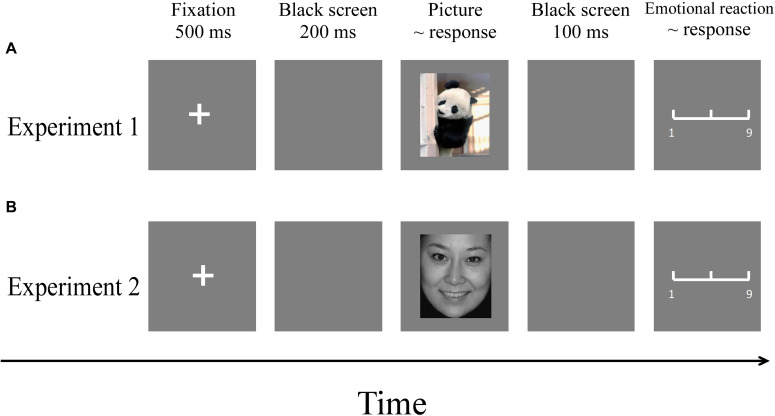
Flowchart describing the experimental designs. **(A)** Procedure of Experiment 1. **(B)** Procedure of Experiment 2. Note: Pictures were selected from the CAPS and the CFAPS that have been previously validated and used in published studies (Bai et al., 2005; Gong et al., 2011; Ma and Zhu, 2014; Wang et al., 2017).

The experiment was carried out in a comfortable and quiet room. Participants partook in both experiments. Pictures were presented in a pseudo-random order using the E-Prime (3.0) program. The order of the two experiments was counterbalanced to control for order effects. The procedures of the two experiments are illustrated in Figure 3.

#### Experiment 1

Each participant carried out the task twice. Participants were randomly given a treatment (painful or control) for the first session and were given the other treatment after a 1-week interval. The order of the two treatments was counterbalanced across participants. All participants were asked to assess their current emotional state based on a 9-point (1 = very unhappy, 5 = neutral, 9 = very happy) Likert scale before the treatment (baseline), 15 min after treatment (pretest), and after the study (posttest) for both the painful and control treatments (Figure 1). Results showed that there were no significant differences (all *p* > 0.05) in emotional states in either the painful or control treatments. These results suggest that the emotional states of the participants were similar for both treatments.

Prior to the experiment, participants were instructed to do a training session in order to get acquainted with the procedure. Two happy pictures, two neutral pictures, and two sad pictures were selected from the CAPS for the training session and were not used in the main experiment. The training session started 15 min after the treatment, and the duration of the training session was about 5 min. Thus, the first formal experiment started at 20 min after the treatment was administered. The duration of the entire experiment was about 15 min.

Each trial involved the following steps:

A fixation cross was presented on a gray screen for a duration of 500 ms. After a 200-ms interval, a picture was presented, during which participants were instructed to respond as accurately and quickly as possible with a key-press (“1,” “2,” or “3”) to judge the emotion type (happy, neutral, or sad) of the picture. The order of key-presses was counterbalanced among participants. The picture remained on the screen until a response was made. After 100 ms, a 9-point emotional assessment scale appeared (1 = very unhappy, 5 = neutral, 9 = very happy), where participants were required to assess their subjective emotional reaction to the picture. The scale disappeared when a response was made. There was an intertrial interval of 500 ms.

#### Experiment 2

Procedures were identical except that the stimuli of the training session and the main experiment were emotional face pictures selected from the CFAPS (Ma and Zhu, 2014; Tan et al., 2018).

Both experiments included two blocks each with a 5-min break between blocks. Each block consisted of 45 trials. The stimuli for each block were pseudo-randomly delivered so that the same emotion type never occurred for three consecutive trials. Each picture was presented three times in each experiment. The order of Experiment 1 and Experiment 2 was counterbalanced among participants, and participants could take a 10-min break between the two experiments.

### Data Analysis

Accuracies (ACCs) and reaction times (RTs) for emotion type judgment, and emotional reactions to pictures were calculated for each participant for each condition. RTs out of the Mean ± 3SD (Experiment 1: 8.78%, Experiment 2: 8.31%) were deleted from the original data. Statistical analyses of the two experiments were performed using SPSS 15.0, using a two-way repeated-measure ANOVA, with two within-participant factors of “treatment” (painful, control) and “emotion” (happy, neutral, sad). The difference values of ACCs, RTs, and emotional reactions between the two treatments (painful–control) were analyzed for three categories of emotional pictures (happy, neutral, sad) using a separate one-way ANOVA for each experiment. The *p*-values of the main effects and interactions were corrected using the Greenhouse–Geisser method. Statistical differences were considered significant at *p* < 0.05.

## Results

**FIGURE 4:**
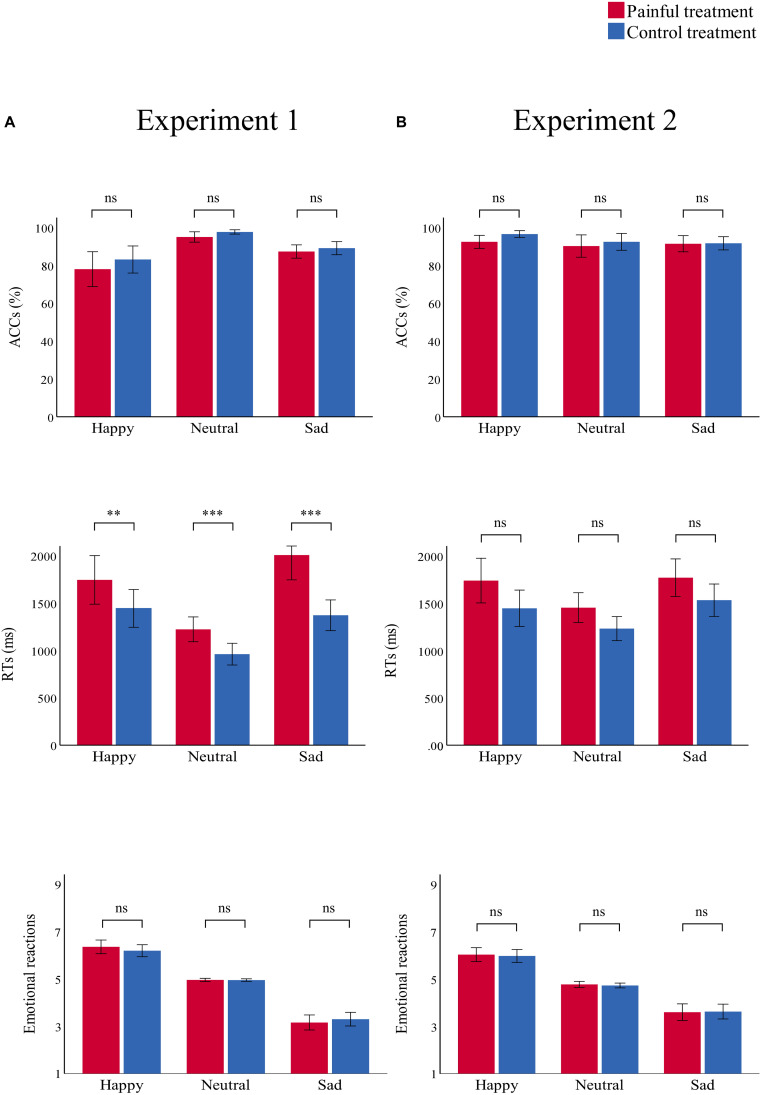
Bar charts representing the results of “treatment” × “emotion” interactions. RTs (top panel), ACCs (middle panel), and emotional reactions (bottom panel) in the painful (red) and control treatment (blue) in **(A)** Experiment 1 and **(B)** Experiment 2. Data are expressed as mean ± SEM. ns: *p* > 0.05; **p < 0.01, ***p < 0.001.

**FIGURE 5:**
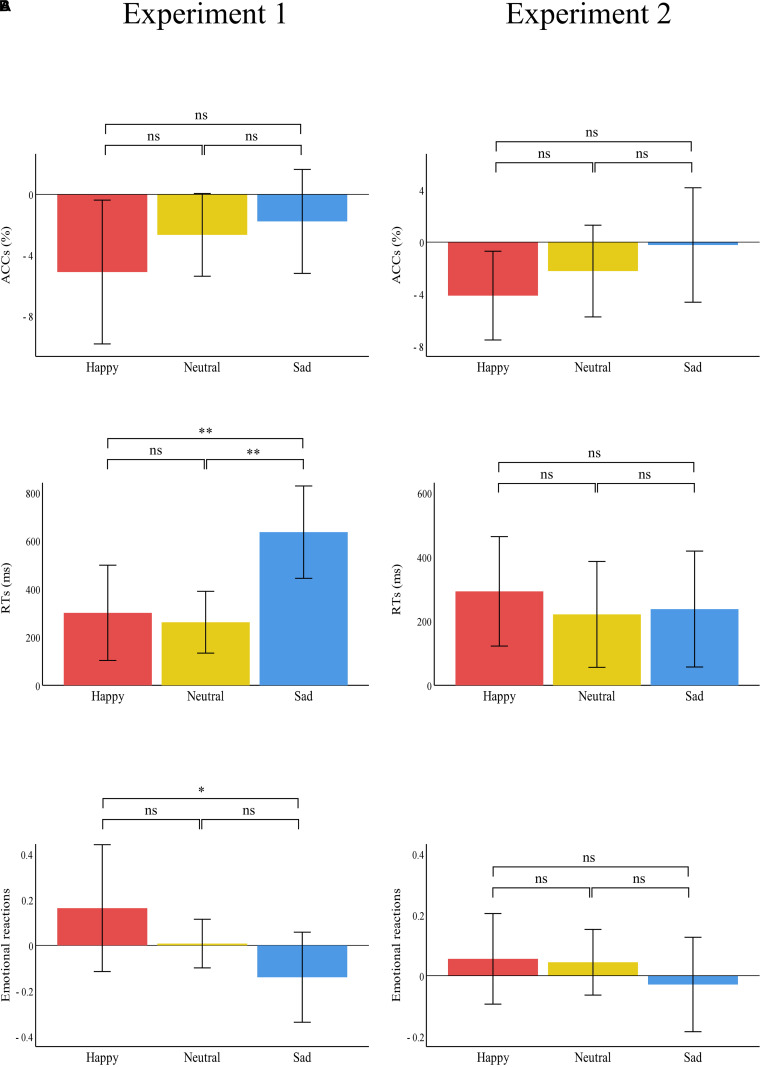
Bar charts representing the results of the one-way ANOVAs of the difference values between the two treatments (painful–control). RTs (top panel), ACCs (middle panel), and emotional reactions (bottom panel) for happy (red), neutral (yellow), and sad (blue) pictures in **(A)** Experiment 1 and **(B)** Experiment 2. Data are expressed as mean ± SEM. ns: *p* > 0.05; *p < 0.05, **p < 0.01.

Accuracies, RTs, and emotional reactions for each condition in the two experiments are summarized in Table 3 and Figures 4, 5.

**TABLE 3:** Summary of two-way repeated-measure ANOVA for Experiment 1 and Experiment 2.

		ACC			RT			Emotional reactions		
		*F*	*p*	η^2^	*F*	*p*	η^2^	*F*	*p*	η^2^
Experiment 1	Treatment	**7.91**	**0.009**	**0.21**	31.21	**<0.001**	0.52	0.02	0.879	<0.01
	Emotion	**19.42**	**<0.001**	**0.58**	25.52	**<0.001**	0.65	**123.39**	**<0.001**	**0.90**
	Treatment × Emotion	0.98	0.371	0.03	12.57	**<0.001**	0.30	2.71	0.085	0.09
Experiment 2	Treatment	3.75	0.063	0.12	12.71	0.001	0.31	0.38	0.543	0.01
	Emotion	1.24	0.295	0.04	16.92	**<0.001**	0.37	**57.70**	**<0.001**	**0.81**
	Treatment × Emotion	1.16	0.322	0.04	0.44	0.636	0.02	0.45	0.628	0.02

*Statistics were obtained using two-way repeated-measure ANOVAs with within-participant factors of treatment and emotion in both Experiment 1 and Experiment 2. df: (2,28). Significant comparisons (p < 0.05) are shown in boldface.*

### Experiment 1

#### ACC

There was a significant main effect of “treatment” [*F*(1,29) = 7.91, *p* = 0.009, η_p_^2^ = 0.21], which indicated that participants were significantly more accurate in the control treatment (90.0 ± 1.3%) than in the painful treatment (86.8 ± 1.7%, *p* = 0.009). There was a significant main effect of “emotion” [*F*(2,28) = 19.42, *p* < 0.001, η_p_^2^ = 0.58], which indicated that participants were significantly more accurate in recognizing neutral emotional scenes (96.3 ± 0.8%) than happy (80.6 ± 3.9%, *p* = 0.001) and sad (88.2 ± 1.5%, *p* < 0.001) scenes; there was no significant difference in ACCs between happy and sad emotional scenes (*p* = 0.074). There was no significant interaction.

#### RT

There was a significant main effect of “treatment” [*F*(1,29) = 31.21, *p* < 0.001, η_p_^2^ = 0.52], which indicated that RTs were significantly shorter in the control treatment (1260.41 ± 67.00) than in the painful treatment (1660.57 ± 95.20, *p* < 0.001). The main effect of “emotion” was significant [*F*(2,28) = 25.52, *p* < 0.001, η_p_^2^ = 0.65]. This result indicated that RTs were significantly shorter for neutral emotional scenes (1093.39 ± 51.44) than for happy (1596.34 ± 101.36, *p* < 0.001) and sad (1691.73 ± 96.02, *p* < 0.001) scenes, while there was no significant difference between happy and sad emotional scenes (*p* = 0.100). There was a significant “treatment” × “emotion” interaction [*F*(2,28) = 12.57, *p* < 0.001, η_p_^2^ = 0.30], which indicated that RTs for recognizing happy, neutral, and sad emotional pictures were significantly shorter in the control (happy: 1445.66 ± 97.58; neutral: 962.20 ± 56.35; sad: 1373.35 ± 79.41) than in the painful treatment (happy: 1747.01 ± 125.36, *p* = 0.004; neutral: 1224.58 ± 63.97, *p* < 0.001; sad: 2010.11 ± 128.61, *p* < 0.001). Furthermore, the one-way ANOVA revealed a significant difference between the three categories of emotional pictures [*F*(2,87) = 5.74, *p* = 0.005, η_p_^2^ = 0.12]. The *post hoc* tests showed that the difference values of RTs for sad emotional scenes (636.76 ± 514.14) were significantly larger than for happy (301.35 ± 530.27, *p* = 0.007) and neutral (262.37 ± 344.34, *p* = 0.003) scenes, while the difference values of RTs for happy and neutral emotional scenes did not differ significantly (*p* = 0.749).

#### Emotional Reaction

There was a significant main effect of “emotion” [*F*(2,28) = 123.39, *p* < 0.001, η_p_^2^ = 0.90], which indicated that emotional reactions to happy scenes (6.28 ± 0.11) were more positive than neutral (4.96 ± 0.01, *p* < 0.001) and sad (3.23 ± 0.14, *p* < 0.001) scenes. Further, emotional reactions to neutral scenes were more positive than sad scenes (*p* < 0.001). No other main effect or interaction was significant.

### Experiment 2

#### ACC

There were no significant main effects or interactions.

#### RT

There was a significant main effect of “treatment” [*F*(1,29) = 12.71, *p* = 0.001, η_p_^2^ = 0.31], which indicated that RTs were significantly shorter in the control (1404.00 ± 72.51) than in the painful treatment (1654.59 ± 87.70, *p* = 0.001). The significant main effect of “emotion” [*F*(2,28) = 16.92, *p* < 0.001, η_p_^2^ = 0.37] indicated that RTs were significantly shorter for neutral emotional faces (1343.08 ± 57.44) than for happy (1593.43 ± 96.60, *p* = 0.001) and sad (1651.37 ± 79.13, *p* < 0.001) faces, while there was no significant difference between happy and sad emotional faces (*p* = 0.263). There was no significant interaction.

#### Emotional Reaction

There was a significant main effect of “emotion” [*F*(2,28) = 57.70, *p* < 0.001, η_p_^2^ = 0.81], which indicated that emotional reactions to happy emotional faces (6.00 ± 0.13) were more positive than neutral (4.75 ± 0.05, *p* < 0.001) and sad (3.61 ± 0.16, *p* < 0.001) faces, and emotional reactions to neutral faces were more positive than sad faces (*p* < 0.001). No other main effect or interaction was significant.

## Discussion

The main goal of this study was to investigate how pain modulates emotional stimuli processing to provide insight into how painful stimuli affect the development of depressive moods. Therefore, we designed two within-subjects experiments using the affective picture paradigm: Experiment 1 tested the effect of pain on responses to emotional scene stimuli, and Experiment 2 tested the same effect using emotional face stimuli. Results of Experiment 1 showed that participants had lower ACCs and longer RTs (especially for sad scenes) for recognizing emotional stimuli in the painful condition, compared to the control condition. In addition, the difference values of RTs between the painful and control conditions for sad scenes were significantly larger than for happy and neutral scenes. In Experiment 2, participants had significantly longer RTs for recognizing emotional face stimuli in the painful condition compared to the control condition.

### Pain Modulates Emotional Stimuli Processing

For both ACCs and RTs, we found that in the painful condition relative to the control condition, participants were less accurate at recognizing emotional scene stimuli and had longer reaction times for recognizing both emotional scenes and faces. These results align with previous research that showed a significant main effect of pain on explicit emotional processing (emotional ratings of emotional stimuli; Godinho et al., 2008). Rosselló et al. (2015) found that compared with healthy participants, patients experiencing pain showed lower startle eyeblink reflex and heart rate variability in all emotional environments, whereby painful stimuli significantly inhibited the processing of emotional stimuli. According to the cognitive-affective model of pain (Eccleston and Crombez, 1999) and previous research (Wieser et al., 2012), the interruptive function of intense pain can distract and divert attention from emotional contents (Ochsner and Gross, 2005). During painful conditions, more attentional resources are allocated to the painful stimuli rather than the emotional stimuli (Reicherts et al., 2013), and thus, the attentional effect of pain weakens the processing of emotional stimuli (Wieser et al., 2012). Accordingly, physical pain shifted individuals’ attention away from the emotional stimuli and thereby reduced the attentional and cognitive resources to process the emotional stimuli. Individuals took significantly longer to attend to and recognize emotional stimuli when experiencing pain than when they had no pain. These results suggest an altered attentional processing of emotional stimuli due to pain, especially regarding sad scene stimuli.

### Pain Modulates Negative Emotional Stimuli Processing

A notable finding was the interaction effect of treatment and emotion observed in Experiment 1, which reflected the larger difference values of RTs for sad scenes between painful and control conditions. These results may suggest attention distraction toward emotional stimuli, especially for sad emotional scenes stimuli when individuals were in pain. The findings support the motivational priming hypothesis (Lang, 1995), as well as partly supporting our own hypothesis. That is, pain can distract people’s attention (Eccleston and Crombez, 1999; Wieser et al., 2012), which was shown in our findings as pain having a strong distracting effect on sad scene stimuli in particular. This result was consistent with previous research that indicated that patients experiencing pain had longer RTs (Duschek et al., 2014) and average fixation durations (Franklin et al., 2018) toward negative emotional stimuli compared with pain-free people. This altered attention processing of negative emotional stimuli may be closely related to the development of depression and may be useful in predicting depression (Armstrong and Olatunji, 2012; Ajilchi and Nejati, 2013). Altered attention processing of negative emotional stimuli may play a vital role in the vicious circle between pain and negative emotions (Duschek et al., 2014). Therefore, pain-induced altered attention processing of sad emotional stimuli might contribute to explaining the effect of painful stimuli on the development of depressive moods.

However, pain did not modulate happy emotional stimuli processing in our study. This result corresponds to an earlier finding of Wieser et al. (2012), which indicated no impact of pain on explicit and implicit emotion processing of happy emotional face stimuli. When emotional stimuli were irrelevant to the present painful stimuli, the processing of emotional stimuli may not be disturbed by pain (Wieser et al., 2012). The contents of the happy face and scene pictures we selected were not directly associated with the current pain experience. As a result, individuals did not pay much attention to happy emotional stimuli. Thus, painful stimuli could not significantly modulate individuals’ responses to happy emotional stimuli.

### The Link Between Pain and Emotional State

Although we observed increased subjective pain intensity in the painful condition, we found that emotional states did not significantly alter with pain. This result suggested that our pain stimuli were not able to induce any negative subjective emotional states during the short duration of the tasks. This outcome is at odds with the pain-depressive mood link, where pain has been shown to significantly augment depressive moods of patients (Hawker et al., 2011; Craig et al., 2013). However, it is possible that the duration and recurrence of pain may play important roles in this link. Moreover, according to the four stages of the pain processing model (Wade et al., 1996; Price, 1999), pain intensity causes pain unpleasantness, which then evokes pain-related emotions, including negative emotions. The pain intensity reported by our participants was moderate, which may not have been sufficiently intense to alter their emotional states negatively.

### Difference Between Responses to Emotional Scenes and Faces

Our results demonstrated different responses to emotional scenes and faces. We found a significant main effect of “treatment” for ACCs for emotional scenes but not for emotional faces. One possible explanation is that emotional stimuli processing was disturbed by pain because of a close association between emotional stimuli and pain experience at the time (Wieser et al., 2012). For example, emotional scenes may have been more relevant than emotional faces to their present pain perception in our study. Moreover, there was a main effect of “emotion” for ACCs for scenes but not for faces. Faces transmit not only emotional information but also social information (Li et al., 2019). From an evolutionary perspective, emotional faces have a survival value in terms of identifying potentially negative information (Straube et al., 2011). In addition, focusing on face stimuli could increase the processing of others’ faces (Li et al., 2020), and people tend to rapidly process emotional faces even in the absence of awareness (Kiss and Eimer, 2010). Therefore, relative to emotional scene stimuli, the processing of emotional face stimuli was not susceptible to pain.

Several limitations of this study should be noted. First, participants may have felt nervous due to their unfamiliarity with the experimental procedure, which may have lowered the accuracy for identifying positive emotional stimuli. Second, all the face stimuli were in gray scale, which may have influenced participants’ responses to emotional face stimuli. Third, we did not examine gender differences. Given that there are differences between men and women in the accuracy of pain detection (Ruben and Hall, 2013) and physiological reaction to emotional stimuli (Šolcová and Lačev, 2017), it is possible that the effects of pain on emotion processing are different in men and women. Future research should include gender as a between-subject factor in the experimental design. Finally, pain and emotion were induced experimentally, so the degree to which the results can be generalized for real-world situations requires further investigation.

## Conclusion

In this study, we employed emotional scene and face pictures to examine how pain modulates responses to emotional stimuli. Results illustrated that recognizing emotional scene stimuli took longer in the painful than control condition, especially for negative emotional scenes. This result supported the notion that pain distracts attentional processing of negative emotional stimuli. Our observation of altered attentional processing of negative emotional stimuli during pain provides insight into understanding how painful stimuli affect the development of depressive moods.

## Data Availability Statement

The datasets presented in this study can be found in online repositories. The names of the repository/repositories and accession number(s) can be found below: https://pan.baidu.com/s/1YeVRSS4gFwWrf0LHdLbCGg (code: 2020).

## Ethics Statement

The studies involving human participants were reviewed and approved by the Local Research Ethics Committee of Chongqing Normal University. The patients/participants provided their written informed consent to participate in this study.

## Author Contributions

WL and JM: concept and design of study. WL and PL: data acquisition, analysis, and interpretation. YH: stimulusmaterials selection and manuscript revision. WL, PL, and JM: drafting the work or revising it critically for importantintellectual content and agreement to be accountable for all aspects of the work in ensuring that questions to theaccuracy or integrity of any part of the work are appropriately investigated and resolved. All authors approved the final the version to be published.

## Conflict of Interest

The authors declare that the research was conducted in the absence of any commercial or financial relationships that could be construed as a potential conflict of interest.
